# Racial, Ethnic, and Immigrant Generational Disparities in Physically Strenuous and Hazardous Work Conditions

**DOI:** 10.1007/s10903-023-01552-8

**Published:** 2023-10-31

**Authors:** Gabino J. Abarca Nava, Anne R. Pebley

**Affiliations:** 1 Fielding School of Public Health and California Center for Population Research, University of California, Los Angeles (UCLA), Los Angeles, CA, USA

**Keywords:** Social determinants, Work conditions, Race and ethnicity, Immigrant generation, Gender, Employment, Strenuous work, Work hazards

## Abstract

Despite the importance of work in adult life, research on the social determinants of health often ignores its effects. We examine race/ethnic, immigrant generational, and gender differentials in exposure to work conditions associated with poor health outcomes, using a nationally-representative sample of adults. On average, Latino 1st generation workers are more exposed to strenuous and hazardous work conditions than other workers, even after adjusting for sociodemographic differences. Exposure is lower for 2nd and 3rd generation Latinos. In contrast, Asian 1st generation men often have the *lowest* exposure levels of all groups and Asian 2nd and 3rd generation men have higher levels of exposure than the first generation, primarily due to intergenerational differences in education. Asian 1st generation women have higher exposures than those in the 2nd or 3rd generation. These results illustrate the importance of considering work conditions in research and policy related to the social determinants of health.

## Introduction

Physical activity is important for health [1, 2]. However, high levels of occupational physical activity (OPA), unlike leisure-time physical activity, appears to be detrimental for many health conditions [3, 4]. High OPA has been associated with musculoskeletal disorders (MSD), functional limitations, and heart disease [5–13]. Physiological pathways through which these associations may work are outlined in the literature [4, 14]. Other exposures, including noise, hazardous chemicals and biological agents, and excess heat or cold can also have detrimental effects on workers’ health [15].

Occupation is closely tied to race, ethnicity, immigration status, gender, and sociodemographic background [16, 17]. Nonetheless, work conditions receive surprisingly little attention in research on social determinants of health [18, 19]. This omission blinds us to a modifiable risk factor through which sociodemographic status, race/ethnicity, gender, and the social structure create health disparities [19].

Latino, especially immigrant, and Black workers are more likely to hold jobs involving higher levels of OPA and/or hazards than others [13, 14, 20, 21]. For example, work-related fatalities are higher for Black and Latino immigrant workers, compared to White workers [22, 23]. Employers have long viewed Latinos, particularly immigrants, as an unlimited supply of cheap, docile, and hardworking manual labor [24–28]. Black workers are also more often steered to jobs involving physical labor than White workers with the same background [27, 29]. Latino and Black workers are also less likely to be promoted out of heavy OPA jobs [30–34]. Thus, they are disproportionately represented in strenuous and hazardous jobs such as construction, meat processing, material moving, health care support, etc. [13, 14, 20].

In this paper, we examine racial, ethnic, and immigrant generational (REIG) disparities in exposure to work conditions associated with poor health outcomes. We extend previous research by: (a) examining immigrant generation, racial, ethnic, and gender differences in work exposures (b) using a nationally-representative sample rather than one from a single industry or locale, and (c) describing the association of sociodemographic characteristics and REIG differences in physical work exposures. We anticipate that Black and Latino men, especially Latino 1st generation men, have the highest exposure of all groups to strenuous and hazardous work. Part of the reason is lower educational attainment than White and many Asian men, but other factors such as discrimination and systemic racism may also be involved. We expect that the patterns are similar for women, although they are less likely overall to hold high OPA jobs than men.

## Methods

We use publically-available data from the 2019 Current Population Survey Annual Social and Economic Supplement (ASEC) (N = 180,101) [35] matched by detailed occupation with job exposures from the U.S. Department of Labor’s Occupational Information Network 26 (O*NET) data [36]. ASEC is based on a sample of the noninstitutionalized US population ages ≥ 16. It collects sociodemographic data, including occupation [35]. O*NET collects data on multiple exposures primarily from samples of job holders for ~ 1000 occupations [37]. O*NET constructs and questionnaires were validated during its design [38] and provide a reasonable level of reliability compared to other survey data [39]. For each ASEC respondent, the matched file contains *average* job exposures from O*NET for each ASEC respondent’s occupation, but not the respondent’s *own* exposures. Thus, differentials in job exposures that we present may be conservative estimates since we cannot measure within-occupation variations (e.g., by race) in exposures. We exclude respondents who were < 18, had no job in the previous year, or were in the Armed Forces (O*NET excludes military occupations), yielding a sample of 89,655 adults.

## Respondent Characteristics

Respondent characteristics, all self-reported, include age (in years), gender (male/female) and race/ethnicity (Latino, non-Hispanic White, non-Hispanic Black, Asian, and Other & Mixed race). Because many Latinos and Asians are immigrants or from immigrant families[40, 41], we subdivided these groups by generation to produce a combined race, ethnicity, and immigrant generational variable (REIG): (1) Latino 1st generation, (2) Latino 2nd generation, (3) Latino 3rd + generation, (4) White, (5) Black, (6) Asian 1st generation, (7) Asian 2nd generation, (8) Asian 3rd + generation, and (9) Other & Mixed race. We define respondents born abroad (except children of US citizens) as 1st generation, those born in the US with at least one foreign-born parent as 2nd, and those born in the US with both parents born in the US as 3rd generation.

To test whether sociodemographic characteristics account for REIG differences in work conditions, we control for educational attainment, specialized occupational training, and region of residence. Educational categories are: < high school, high school or equivalent, some college but no degree, vocational associate’s degree, academic associate’s degree, and ≥ bachelor’s degree. Specialized training indicates whether the respondent has a certificate or industry license. Both the prevalence of occupations and distribution of REIG groups vary considerably by region. We include whether the respondent lives in the Northeast, Midwest, South or West. We also include: self-rated health status (excellent, very good or good health vs. fair or poor health) and marital status (currently married vs. not married), which may also affect occupational choice.

## Physical Job Exposures

We focus on three types of job exposures for respondents’ longest-held occupation during the year prior to interview. The specific O*NET questions used to measure these exposures are provided in Online Resource 1. The first is the *level of general occupational physical activity* (i.e., OPA) which has been associated with MSD [14] and cardiovascular disease [5, 42]. This index combines four O*NET measures: general level of physical activity, importance of physical activity to the job, time (in categories) running or walking, and time keeping or regaining balance. The second is *specific work conditions associated with MSD*: posture, force, repetition, and vibration, from an extensive review by Andrasfay et al. [14]. The posture index includes five O*NET items on time spent standing, sitting, kneeling, crouching, stooping, crawling, bending, twisting, and working in cramped spaces and awkward postures. The force, repetition, and vibration indices all include only one item. The force index is time spent using hands to handle, control, or feel objects, tools, or controls. The repetition index is time spent making repetitive motions. The vibration index is frequency of exposure to whole-body vibration. The third type is *exposure to occupational hazards* and includes six items on using poles, scaffolding, catwalks, ladders > 8 feet, hazardous equipment, contaminants, radiation, and very hot/cold temperatures. These occupational hazards are associated with health conditions from injuries to cancers and other diseases [43, 44].

All O*NET items included have 5 response categories, except for general physical activity which has 7. Responses for each item were standardized to range from 0 (lowest) to 1 (highest). To construct composite scales for indices containing multiple items, we average standardized responses across items in the scale.

## Analysis

After describing the sample, we use multivariable statistical models to investigate the associations between REIG and work conditions, controlling only for age. Next, we adjusted these models for sociodemographic characteristics which may account for these associations. All models are estimated separately by gender, using Stata 17.0 [45].

## Results

Table 1 shows the sociodemographic variables by REIG. Average age is similar for all groups, except for 2nd generation Latinos and Asians who are younger than others. There are more men than women, because of gender differences in labor force participation—except for Black respondents for whom there are more women than men, most likely because of higher incarceration and unemployment among Black men and higher labor force participation of Black women compared to other groups [46–48].

The most striking difference by REIG is educational attainment. Latinos, particularly 1st generation Latinos, have substantially less education than the others. Asians, on the other hand, have more education than others. Divergence in educational attainment of Latino and Asian immigrants is due to substantial differences in their histories of, and current options for, immigration to the US, and to educational opportunities in home countries [49–51]. Specialized occupational training is most common among White respondents and least common among 1st and 2nd generation Latinos and 1st generation Asians.

REIG groups are geographically concentrated in different regions of the US, which may affect the job market conditions and the amount of discrimination that they face. Latino, Asian, and Other & Mixed respondents live primarily in the West and South, whereas Black respondents live predominantly in the South. White participants are fairly evenly distributed across regions.

Most respondents in the sample report being in excellent, very good, or good health. 1st generation Latinos and Asians as well as White respondents have the highest percent married. “Never married” status is most frequent for 2nd generation Latinos and Asians. Black and Other & Mixed respondents also had relatively high proportions never married.

The mean and standard deviation of the outcome variables are shown in Table 2. First generation Latinos’ scores for strenuous and hazardous work are higher than all other groups. In general, Latinos’ scores are higher, and Asians’ scores are generally the same as, or lower than, other race/ethnic groups.

## Multivariable Analysis Results

The first goal is to determine whether Latino workers, particularly immigrants, are exposed to more strenuous and hazardous conditions than other workers. To answer this question, we examine unadjusted values by estimating ordinary least squares (OLS) regressions for each work conditions index by REIG controlling only for age. The second goal is to determine whether sociodemographic characteristics (education, occupational training, region, health status, and marital status) accounted for observed differences by REIG. To do so, we re-estimated the OLS models adding these characteristics and refer to these results as adjusted values. The results for the regression models are in Online Resource 2.

**Fig. 1:**
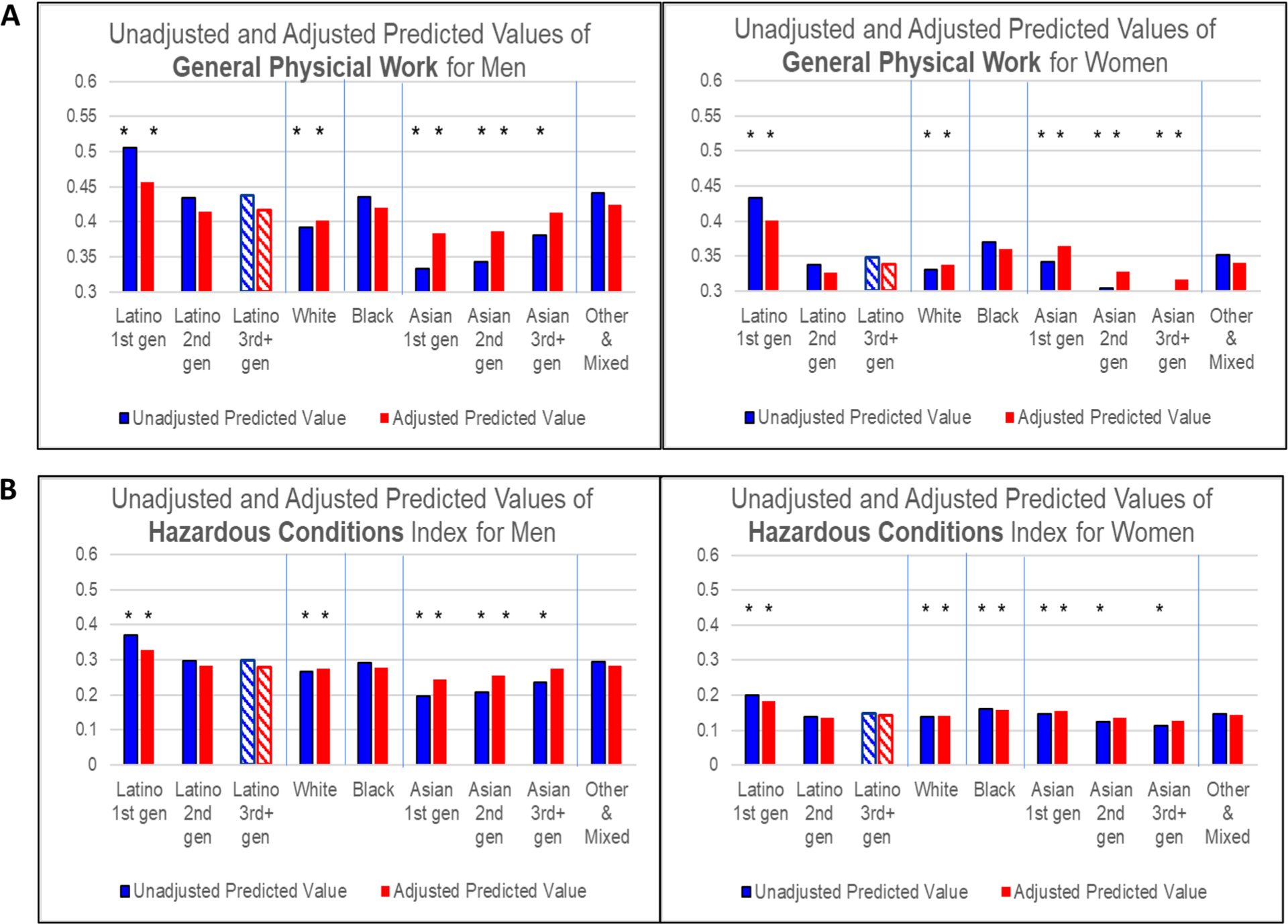
**A** Predicted Values of General Physical Work Levels for Men and Women by Race/Ethnicity and Immigrant Generation. * Indicates that the estimated coefficient on which this predicted value is based is statistically significantly different from Latino 3rd gen respondents of the same gender at p < .001. To indicate that the Latino 3rd gen category is the comparison group in the multilevel models, the bars for this group are cross-hatched rather than solid. **B** Predicted Values of Frequency of Exposure to Hazardous Work Conditions for Men and Women by Race/Ethnicity and Immigrant Generation. * Indicates that the estimated coefficient on which this predicted value is based is statistically significantly different from Latino 3rd gen respondents of the same gender at p < .001. To indicate that the Latino 3rd gen category is the comparison group in the multilevel models, the bars for this group are cross-hatched rather than solid

**Fig. 2:**
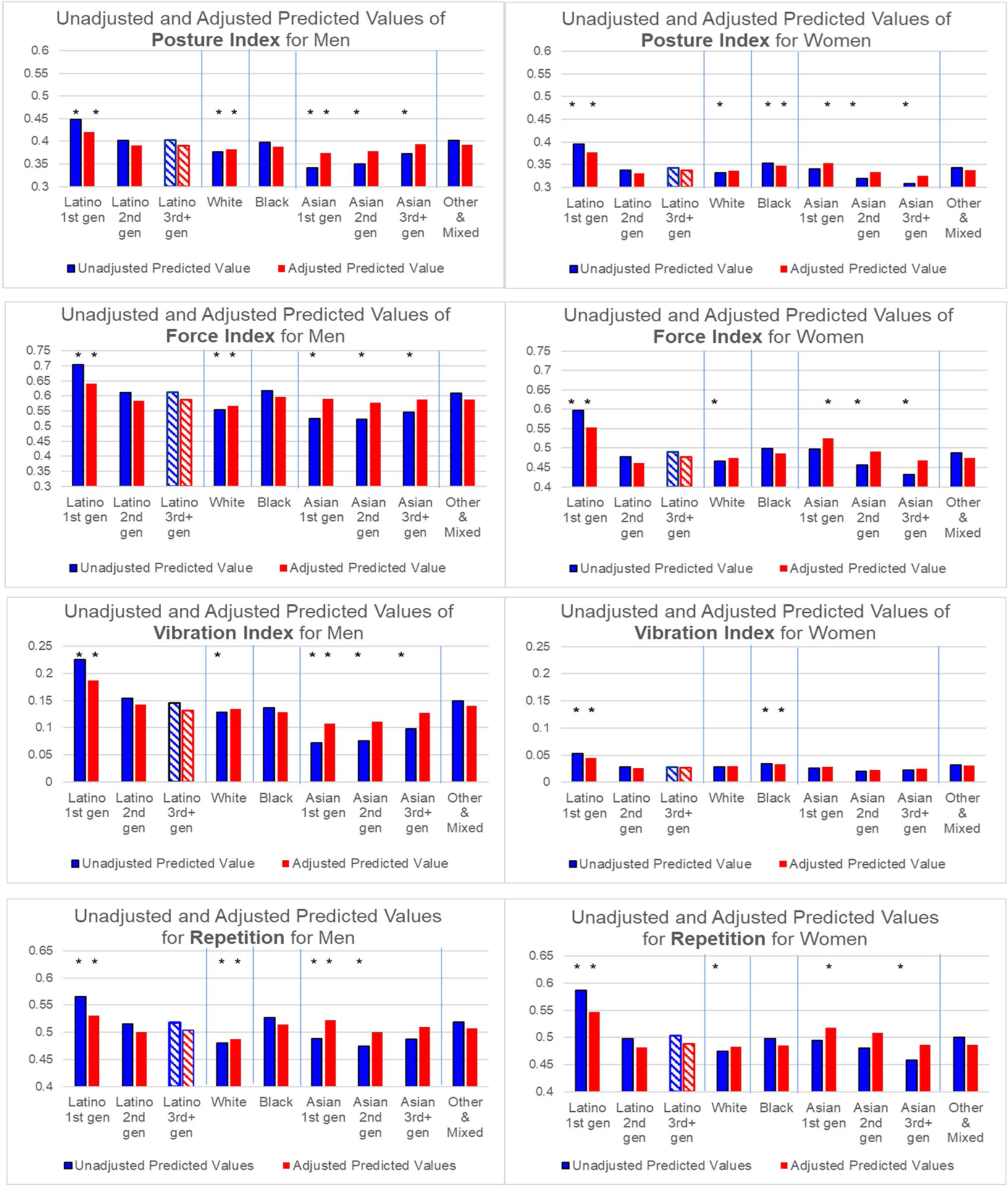
Predicted Values of MSC-Related Work Conditions for Men and Women by Race/Ethnicity and Immigrant Generation. * Indicates that the estimated coefficient on which this predicted value is based is statistically significantly different from Latino 3rd gen respondents of the same gender at p < .001. To indicate that the Latino 3rd gen category is the comparison group in the multilevel models, the bars for this group are cross-hatched rather than solid

We use these regression results to calculate predicted values of each dependent variable by REIG and gender, holding all other independent variables constant at their observed values. These predicted values are graphed in Figs. 1 and 2. The Y-axes depend on the measure’s distribution and cannot be compared across different measures. The asterisks indicate that a REIG group’s coefficient in the model was statistically significantly different from the omitted group (Latino 3 + gen) at p < 0.001 (shown as a striped bar).

Examination of the results in Figs. 1A, B and 2 reveal a consistent picture of REIG and gender differences in work conditions, despite the wide range of measures examined. This pattern for the unadjusted models is shown in the bar on the left of each pair of bars in each graph and summarized below. These results were confirmed by t-tests of coefficients between each pair of REIG categories. Below we only report statistically significant differences based on the t-tests at p < 0.001.

- Men are exposed to higher levels of strenuous and hazardous work than women. Nonetheless, 1st generation Latino women, on average, hold jobs that have equal or higher levels of general physical work than White, and Asian 1st and 2nd generation *men*.
- Latino 1st generation men and women typically have higher levels of strenuous and hazardous work than all other REIG groups of the same gender. The predicted values are significantly lower for 2nd and 3rd + generation Latino workers compared to 1st generation Latinos, but higher than for other groups, such as Asian and White workers.
- Asian 1st generation men often have the *lowest* levels of all groups on measures of strenuous and hazardous work (except for force and repetition). In contrast to Latinos, for Asian men, strenuous and hazardous work is *more* common in the 2nd and 3rd + generation groups than in the 1st generation group. Unlike Asian men, 1st generation Asian women have higher values on many of the measures compared to 2nd and 3rd + generation Asian women.
- Whites, on average, do less strenuous and hazardous work than others, while Black men’s level is typically about the same as 2nd and 3rd + generation Latino men. This is also true for Black women, but differences are smaller.
- The Mixed & Other group is similar to Black workers, especially for men.

Why are the experiences by generation so different for Latinos and Asians? Large differences in sociodemographic characteristics between Latino and Asian immigrants to the US may be part of the reason. To test this hypothesis, we re-estimated the OLS regressions, adding education, specialized occupational training, region, marital status, and self-reported health to the model including age. The predicted values derived from these adjusted models are shown in the graphs on the right side of each pair of bars in Figs. 1A, B and 2. The OLS coefficients (see Online Resource 2) indicate that educational attainment is consistently, significantly related to strenuous and hazardous work exposures; other sociodemographic variables are significant only for some outcome measures.

For men, controlling for these variables eliminates much of the generational differences among Asians, suggesting that low rates of strenuous and hazardous work among 1st generation Asians is partly attributable to their higher educational attainment, compared to other Asian generations and other groups. The gap between Latino and Asian men on all measures is narrower in the adjusted results. Nonetheless, the levels of strenuous and hazardous work for Latino men, especially 1st generation men, on average, remain higher in the adjusted results than for Asian men.

For women, sociodemographic controls narrow the gap between Latino and Asian workers on hazardous exposure, but not for general physical work. For most MSD-related measures, the differences between Asian 1st generation women and Latino 3rd generation women increase in the adjusted results.

Generational differences remain sizeable for Asian and Latino women after adjustment: 1st generation women remain more exposed to strenuous work on many measures compared to 2nd and 3rd generation women of the same race/ethnicity.

The adjusted results also reveal that Black workers have significantly higher exposure to difficult work conditions than Whites (except for vibration and hazardous conditions for men and repetition for women), although some differences are small. Adjusted exposures for Black, Asian 3rd generation, and Latino 3rd generation workers are similar on most indicators.

## Discussion

Our results demonstrate that Latino 1st generation workers are more exposed to strenuous and hazardous work than others, even after adjusting for sociodemographic differences. All immigrants must adapt to a new social and work environment, but undocumented Latino immigrants face additional roadblocks. Both undocumented workers and those with liminal legal status, such as Deferred Action for Childhood Arrivals (DACA) and Temporary Protected Status (TPS) [52], are more vulnerable. They are more likely to have informal and unregulated jobs, to not report safety violations and injuries for fear of being fired or deported, and to be unfamiliar with US labor laws and regulations [53–57]. ASEC, like other nationally-representative surveys, does not include information on documentation status because of potential risks to undocumented respondents. Therefore, we cannot determine whether the higher exposures to strenuous and hazardous work for Latino 1st generation workers are concentrated among the undocumented. Nonetheless, Latino 2nd and 3rd generation workers (US born citizens) also are more exposed to strenuous work than Whites. Previous research suggests that at least part of the reason is discrimination faced by Latino (and Black) workers in the labor market and on the job [30, 58, 59].

The contrasting generational patterns among Asian and Latino immigrants may be due, in part, to changes in the origins and characteristics of Asian immigrants in recent years. Both the Latino and Asian populations in the US vary considerably by national origin and socioeconomic status [60]. The origins of both groups have been changing, but changes in the Asian population have been larger. For example, in 1980, only 8 percent of the foreign-born population was Indian [61] compared with nearly 20 percent in 2021 [62], due primarily to the immigration of high-tech/highskilled workers.

This study has several limitations. First, O*NET measures are limited in some areas and its design and question wording could be improved [63–66]. Second, O*NET data reflect the *average* experience of workers in each occupation. No information is available on variation in physical activity *within* occupations. Thus, our results may underrepresent disparities between Latino 1st generation workers and others, if these workers do more physically challenging tasks *within* their occupations than other workers. Third, ASEC does not include several potentially important predictors of occupation for immigrants, including English speaking ability, duration of time in the US, previous work experience, and documentation status.

Our results are important for at least three reasons. First, they demonstrate the need for effective safety and health programs and practices in workplaces employing vulnerable workers, such as Latino 1st generation immigrants. Creating environments in which immigrant workers can report violations without fear of retribution is crucial but difficult [67, 68]. Lasting improvements in immigrant worker safety would benefit from a more permanent legal status for undocumented and liminally legal workers as well as increasing immigrant worker protection and active occupational safety education. Changes to guestworker visa programs are also essential. Temporary work visas tie workers to individual employers: these visas depend continued employment by a particular employer. Thus, employers have de facto deportation power over temporary immigrant workers and workers have little ability to complain about, or report, poor work conditions [69, 70]. Second, health care professionals need a concrete understanding of the workplace exposures encountered by their patients. Although exposures vary considerably within each REIG group, our results provide a starting point for discussion with patients, particularly Latino immigrants, about the physical work conditions they may face. Third, these results demonstrate the importance of incorporating work conditions in research and policy on social determinants of health. They also contribute to the literature on the “Hispanic paradox,” i.e., Latinos’ survival advantage compared to Whites, despite a lower average socioeconomic status [71, 72]. In contrast to mortality, disability rates among older adults are *higher* for Latinos than Whites [13, 73]. Our results suggest that work conditions may be a contributing factor.

## Supplementary Material

Supp 1

Supp 2

## Figures and Tables

**Table 1:** Sociodemographic characteristics by race, ethnicity and immigrant generation (REIG) (N = 89,655), from CPS/ASEC 2019 data

Variable	Latino					Asian				
	1st Gen	2nd Gen	3rd+ Gen	White	Black	1st Gen	2nd Gen	3rd+ Gen	Other & Mixed	All
	% (n)	% (n)	% (n)	% (n)	% (n)	% (n)	% (n)	% (n)	% (n)	% (n)
Age (mean (SD))	43.28 (12.36)	33.01 (12.54)	38.49 (14.15)	43.46 (14.81)	42.83 (14.35)	44.24 (12.89)	35.48 (13.09)	45.56 (15.31)	39.31 (15.00)	42.44 (14.57)
Gender										
Male	58.56% (4707)	50.94% (2029)	50.17% (2564)	52.10% (28,518)	44.18% (4091)	52.26% (2027)	52.51% (649)	50.09% (280)	50.05% (1430)	51.64% (46,295)
Female	41.44% (3331)	49.06% (1954)	49.83% (2547)	47.90% (26,214)	55.82% (5169)	47.74% (1852)	47.49% (587)	49.91% (279)	49.95% (1427)	48.36% (43,360)
Education										
Less than high school	37.75% (3034)	11.45% (456)	10.06% (514)	5.73% (3138)	7.59% (703)	6.63% (257)	5.58% (69)	2.33% (13)	8.72% (249)	9.41% (8433)
High school or equivalent	31.36% (2521)	30.13% (1200)	31.95% (1633)	23.96% (13,112)	31.49% (2916)	17.43% (676)	13.43% (166)	15.56% (87)	31.19% (891)	25.88% (23,202)
Some college, no degree	10.13% (814)	25.63% (1021)	23.26% (1189)	17.17% (9398)	20.65% (1912)	8.61% (334)	17.48% (216)	16.64% (93)	23.03% (658)	17.44% (15,635)
Occupation/Vocational (AA)	2.46% (198)	4.87% (194)	4.54% (232)	5.24% (2866)	4.49% (416)	2.84% (110)	3.07% (38)	3.94% (22)	5.22% (149)	4.71% (4225)
Academic associate degree	2.94% (236)	6.65% (265)	6.34% (324)	6.41% (3508)	6.45% (597)	3.87% (150)	3.96% (49)	5.72% (32)	6.27% (179)	5.96% (5340)
Bachelor’s degree or more	15.36% (1235)	21.27% (847)	23.85% (1219)	41.49% (22,710)	29.33% (2716)	60.63% (2352)	56.47% (698)	55.81% (312)	25.59% (731)	36.61% (32820)
Certificate/industry license										
No	89.74% (7213)	82.25% (3276)	80.14% (4096)	72.55% (39,710)	79.01% (7316)	80.36% (3117)	78.48% (970)	79.60% (445)	78.05% (2230)	76.26% (68,373)
Yes	10.26% (825)	17.75% (707)	19.86% (1015)	27.45% (15,022)	20.99% (1944)	19.64% (762)	21.52% (266)	20.39% (114)	21.95% (627)	23.74% (21,282)
Region										
Northeast	12.52% (1006)	8.54% (340)	12.44% (636)	18.38% (10,058)	13.54% (1254)	20.65% (801)	18.77% (232)	6.44% (36)	8.19% (234)	16.28% (14,597)
Midwest	9.77% (785)	10.22% (407)	10.15% (519)	24.76% (13,554)	13.84% (1282)	12.43% (482)	9.55% (118)	4.65% (26)	14.53% (415)	19.62% (17,588)
South	38.94% (3130)	31.01% (1235)	32.11% (1641)	32.70% (17,897)	64.55% (5977)	23.36% (906)	20.71% (256)	8.77% (49)	26.46% (756)	35.52% (31,847)
West	38.78% (3117)	50.24% (2001)	45.29% (2315)	24.16% (13,223)	8.07% (747)	43.57% (1690)	50.97% (630)	80.14% (448)	50.82% (1452)	28.58% (25,623)
Health status										
Excellent/very good/good	92.35% (7423)	93.42% (3721)	92.96% (4751)	93.86% (51,370)	91.31% (8455)	94.46% (3664)	95.47% (1180)	93.56% (523)	90.44% (2584)	93.33% (83,671)
Fair/poor health	7.65% (615)	6.58% (262)	7.04% (360)	6.14% (3362)	8.69% (805)	5.54% (215)	4.53% (56)	6.44% (36)	9.56% (273)	6.67% (5984)
Marital status										
All married	62.98% (5062)	35.90% (1430)	44.81% (2290)	61.54% (33,683)	37.84% (3504)	73.99% (2870)	42.07% (520)	55.28% (309)	41.79% (1194)	56.73% (50,862)
Widowed/divorced/seperated	13.46% (1082)	9.26% (369)	13.21% (675)	13.81% (7560)	17.67% (1636)	8.02% (311)	5.50% (68)	9.12% (51)	14.56% (416)	13.57% (12,168)
Never married	23.56% (1894)	54.83% (2184)	41.99% (2146)	24.65% (13489)	44.49% (4120)	17.99% (698)	52.43% (648)	35.60% (199)	43.65% (1247)	29.70% (26,625)
Total	8038	3983	5111	54732	9260	3879	1236	559	2857	89,655

**Table 2:** Measures of physically strenuous and hazardous work conditions by race, ethnicity and immigrant generation (REIG) (N = 89,655)

Variable	Latino					Asian				
	1st Gen	2nd Gen	3rd+ Gen	White	Black	1st Gen	2nd Gen	3rd+ Gen	Other and mixed	All
	Mean (SD)	Mean (SD)	Mean (SD)	Mean (SD)	Mean (SD)	Mean (SD)	Mean (SD)	Mean (SD)	Mean (SD)	Mean (SD)
General physical index	0.48 (0.15)	0.39 (0.16)	0.40 (0.16)	0.36 (0.16)	0.30 (0.16)	0.34 (0.17)	0.33 (0.16)	0.33 (0.17)	0.40 (0.16)	0.38 (0.17)
Posture index	0.43 (0.10)	0.37 (0.10)	0.37 (0.10)	0.35 (0.10)	0.37 (0.10)	0.34 (0.09)	0.34 (0.09)	0.34 (0.10)	0.37 (0.10)	0.36 (0.10)
Force index	0.66 (0.20)	0.56 (0.21)	0.56 (0.21)	0.51 (0.22)	0.55 (0.21)	0.51 (0.21)	0.50 (0.21)	0.48 (0.21)	0.55 (0.21)	0.53 (0.22)
Vibration index	0.15 (0.19)	0.09 (0.15)	0.09 (0.15)	0.08 (0.14)	0.08 (0.13)	0.05 (0.10)	0.05 (0.10)	0.06 (0.12)	0.09 (0.15)	0.09 (0.15)
Repetition index	0.57 (0.17)	0.52 (0.16)	0.51 (0.16)	0.48 (0.17)	0.51 (0.16)	0.49 (0.17)	0.48 (0.16)	0.47 (0.16)	0.51 (0.16)	0.49 (0.17)
Hazardous conditions index	0.30 (0.18)	0.22 (0.17)	0.22 (0.17)	0.21 (0.17)	0.22 (0.16)	0.17 (0.14)	0.17 (0.14)	0.17 (0.16)	0.22 (0.17)	0.22 (0.17)
