# Supplementary material for: Racial, Ethnic, and Immigrant Generational Disparities in Physically Strenuous and Hazardous Work Conditions
Source: J Immigr Minor Health. Author manuscript; Available in PMC 2024 Apr 1. (PMC10937783; doi:10.1007/s10903-023-01552-8)
Supplement: Supp 1 [file NIHMS1944658-supplement-Supp_1.pdf]

Online Resource 2

Paper Title: “Racial, Ethnic, and Immigrant Generational Disparities in Physically Strenuous and Hazardous Work Conditions”

Journal: Journal of Immigrant and Minority Health

Caption: This file contains tables of the regression results from which the predicted values in Figures 1A and B and Figure 2 in the paper were derived.

Online Resource Table S1. Regression Results with General Physical Index as Dependent Variable

**General Physical Index**												
	Male Unadjusted			Male Adjusted			Female Unadjusted			Female Adjusted		
	**Coefficient**	**t**	**P>t**	**Coefficient**	**t**	**P>t**	**Coefficient**	**t**	**P>t**	**Coefficient**	**t**	**P>t**
**Race, Ethnicity & Immigration Gen**												
Reference (Latino, USB, 3rd+)												
Latino, USB, 2nd	-0.003	-0.68	0.499	-0.003	-0.70	0.484	-0.010	-2.28	0.023	-0.012	-2.98	0.003
Latino, FB, 1st	0.068	16.62	0.000	0.039	10.63	0.000	0.085	21.63	0.000	0.062	16.61	0.000
NH White	-0.046	-13.21	0.000	-0.015	-5.01	0.000	-0.017	-5.47	0.000	-0.002	-0.63	0.529
NH Black	-0.002	-0.59	0.554	0.003	0.70	0.482	0.022	5.97	0.000	0.021	6.15	0.000
Asian, USB, 3rd +	-0.058	-5.51	0.000	-0.004	-0.39	0.695	-0.059	-6.30	0.000	-0.022	-2.53	0.011
Asian, USB, 2nd	-0.095	-12.93	0.000	-0.032	-4.93	0.000	-0.044	-6.49	0.000	-0.012	-1.82	0.069
Asian, FB, 1st	-0.105	-21.21	0.000	-0.034	-7.71	0.000	-0.007	-1.54	0.124	0.025	5.85	0.000
Other & Mixed	0.003	0.47	0.636	0.007	1.42	0.155	0.003	0.60	0.546	0.002	0.41	0.680
**Age**	-0.001	-14.27	0.000	0.000	-0.82	0.410	-0.001	-14.02	0.000	0.000	-4.77	0.000
**Education**												
Reference (Less than HS)												
High school or equivalent				-0.018	-7.15	0.000				-0.054	-19.07	0.000
Some college, no degree				-0.068	-24.65	0.000				-0.086	-29.11	0.000
Occupation/Vocational training (AA)				-0.038	-9.74	0.000				-0.078	-19.65	0.000
Academic associate degree				-0.092	-24.46	0.000				-0.108	-30.11	0.000
Bachelor's degree or more				-0.210	-81.14	0.000				-0.165	-58.76	0.000
**Certificate/Industry License**												
Reference (No)												
Yes				0.012	6.92	0.000				0.042	26.22	0.000
**Region**												
Reference (Northeast)												
Midwest				0.005	2.00	0.045				0.004	1.89	0.059
South				0.002	1.02	0.306				-0.004	-2.22	0.026
West				-0.004	-1.99	0.046				-0.003	-1.29	0.197
**Health Status**												
Reference (Excellent/Very Good/Good)												
Fair/Poor Health				0.004	1.55	0.122				0.013	4.89	0.000
**Marital Status**												
Reference (All Married)												
Widowed/Divorced/Seperated				0.012	5.28	0.000				0.017	9.08	0.000
Never Married				0.010	5.33	0.000				0.024	13.55	0.000
**Constant**	0.471	121.10	0.000	0.506	108.06	0.000	0.378	107.17	0.000	0.434	91.92	0.000
Number of Cases	46,295						43,360					

Online Resource Table S2. Regression Results with Hazardous Conditions Index as Dependent Variable

**Hazardous Conditions Index**	Male Unadjusted			Male Adjusted			Female Unadjusted			Female Adjusted		
	**Coefficient**	**t**	**P>t**	**Coefficient**	**t**	**P>t**	**Coefficient**	**t**	**P>t**	**Coefficient**	**t**	**P>t**
**Race, Ethnicity & Immigration Gen**												
Reference (Latino, USB, 3rd+)												
Latino, USB, 2nd	0.001	0.16	0.874	0.003	0.60	0.548	-0.007	-2.29	0.022	-0.008	-2.52	0.012
Latino, FB, 1st	0.074	16.18	0.000	0.047	11.21	0.000	0.053	18.88	0.000	0.040	14.12	0.000
NH White	-0.031	-8.04	0.000	-0.006	-1.72	0.085	-0.007	-3.36	0.001	-0.002	-0.75	0.451
NH Black	-0.006	-1.31	0.190	-0.002	-0.42	0.676	0.014	5.22	0.000	0.014	5.25	0.000
Asian, USB, 3rd +	-0.063	-5.38	0.000	-0.007	-0.66	0.512	-0.034	-5.09	0.000	-0.018	-2.69	0.007
Asian, USB, 2nd	-0.091	-11.12	0.000	-0.025	-3.41	0.001	-0.023	-4.65	0.000	-0.008	-1.66	0.096
Asian, FB, 1st	-0.103	-18.57	0.000	-0.037	-7.29	0.000	0.000	-0.07	0.944	0.012	3.70	0.000
Other & Mixed	-0.002	-0.32	0.753	0.002	0.44	0.660	0.001	0.22	0.827	0.001	0.16	0.876
**Age**	0.000	3.10	0.002	0.000	4.40	0.000	0.000	-0.61	0.544	0.000	2.24	0.025
**Education**												
Reference (Less than HS)												
High school or equivalent				0.006	1.94	0.052				-0.026	-12.12	0.000
Some college, no degree				-0.054	-17.13	0.000				-0.048	-21.53	0.000
Occupation/Vocational training (AA)				-0.001	-0.19	0.846				-0.043	-14.53	0.000
Academic associate degree				-0.072	-16.67	0.000				-0.055	-20.29	0.000
Bachelor's degree or more				-0.189	-63.39	0.000				-0.076	-36.19	0.000
**Certificate/Industry License**												
Reference (No)												
Yes				0.023	11.94	0.000				0.010	8.28	0.000
**Region**												
Reference (Northeast)												
Midwest				0.009	3.60	0.000				0.009	5.20	0.000
South				0.010	4.36	0.000				0.001	0.86	0.392
West				-0.003	-1.16	0.248				0.001	0.47	0.636
**Health Status**												
Reference (Excellent/Very Good/Good)												
Fair/Poor Health				0.003	0.90	0.370				0.004	1.79	0.074
**Marital Status**												
Reference (All Married)												
Widowed/Divorced/Seperated				0.007	2.70	0.007				0.007	5.21	0.000
Never Married				-0.025	-12.01	0.000				0.004	2.95	0.003
**Constant**	0.290	66.98	0.000	0.341	63.12	0.000	0.148	58.17	0.000	0.183	51.38	0.000
Number of Cases	46,295						43,360					

Online Resource Table S3. Regression Results with Posture Index as Dependent Variable

Posture Index												
	Male Unadjusted			Male Adjusted			Female Unadjusted			Female Adjusted		
	**Coefficient**	**t**	**P>t**	**Coefficient**	**t**	**P>t**	**Coefficient**	**t**	**P>t**	**Coefficient**	**t**	**P>t**
**Race, Ethnicity & Immigration Gen**												
Reference (Latino, USB, 3rd+)												
Latino, USB, 2nd	0.000	-0.13	0.897	0.000	0.05	0.960	-0.005	-1.99	0.046	-0.006	-2.63	0.009
Latino, FB, 1st	0.046	17.47	0.000	0.029	12.22	0.000	0.054	24.79	0.000	0.040	19.48	0.000
NH White	-0.026	-11.85	0.000	-0.009	-4.35	0.000	-0.010	-5.80	0.000	-0.001	-0.77	0.439
NH Black	-0.006	-2.06	0.040	-0.003	-1.29	0.196	0.011	5.61	0.000	0.011	5.88	0.000
Asian, USB, 3rd +	-0.031	-4.57	0.000	0.003	0.52	0.605	-0.034	-6.53	0.000	-0.012	-2.49	0.013
Asian, USB, 2nd	-0.052	-11.10	0.000	-0.013	-3.07	0.002	-0.023	-6.14	0.000	-0.004	-1.03	0.305
Asian, FB, 1st	-0.061	-18.94	0.000	-0.017	-5.98	0.000	-0.002	-0.88	0.380	0.016	6.80	0.000
Other & Mixed	-0.001	-0.19	0.847	0.002	0.61	0.539	0.001	0.48	0.630	0.001	0.38	0.706
**Age**	0.000	-10.26	0.000	0.000	-0.65	0.514	0.000	-8.24	0.000	0.000	-0.84	0.403
**Education**												
Reference (Less than HS)												
High school or equivalent				-0.005	-3.29	0.001				-0.029	-18.41	0.000
Some college, no degree				-0.040	-22.32	0.000				-0.047	-29.27	0.000
Occupation/Vocational training (AA)				-0.009	-3.53	0.000				-0.043	-19.96	0.000
Academic associate degree				-0.051	-20.89	0.000				-0.059	-30.01	0.000
Bachelor's degree or more				-0.124	-73.64	0.000				-0.094	-60.78	0.000
**Certificate/Industry License**												
Reference (No)												
Yes				0.012	10.53	0.000				0.019	22.04	0.000
**Region**												
Reference (Northeast)												
Midwest				0.002	1.11	0.267				0.003	2.38	0.017
South				0.003	2.36	0.018				-0.002	-1.62	0.106
West				-0.003	-2.49	0.013				-0.002	-1.65	0.099
**Health Status**												
Reference (Excellent/Very Good/Good)												
Fair/Poor Health				0.003	1.68	0.094				0.008	5.48	0.000
**Marital Status**												
Reference (All Married)												
Widowed/Divorced/Seperated				0.008	5.13	0.000				0.009	8.90	0.000
Never Married				0.001	0.86	0.389				0.010	10.22	0.000
**Constant**	0.418	167.02	0.000	0.441	144.06	0.000	0.352	180.56	0.000	0.387	149.05	0.000
Number of Cases	46,295						43,360					

Online Resource Table S4. Regression Results with Force Index as Dependent Variable

**Force Index**												
	Male Unadjusted			Male Adjusted			Female Unadjusted			Female Adjusted		
	**Coefficient**	**t**	**P>t**	**Coefficient**	**t**	**P>t**	**Coefficient**	**t**	**P>t**	**Coefficient**	**t**	**P>t**
**Race, Ethnicity & Immigration Gen**												
Reference (Latino, USB, 3rd+)												
Latino, USB, 2nd	-0.002	-0.30	0.767	-0.002	-0.33	0.739	-0.014	-2.35	0.019	-0.016	-2.95	0.003
Latino, FB, 1st	0.091	16.66	0.000	0.054	10.98	0.000	0.107	21.25	0.000	0.076	15.87	0.000
NH White	-0.059	-12.92	0.000	-0.021	-5.04	0.000	-0.024	-5.98	0.000	-0.002	-0.47	0.640
NH Black	0.005	0.85	0.397	0.010	1.92	0.055	0.009	2.01	0.045	0.009	2.08	0.038
Asian, USB, 3rd +	-0.068	-4.84	0.000	0.002	0.15	0.883	-0.059	-4.91	0.000	-0.009	-0.77	0.439
Asian, USB, 2nd	-0.089	-9.15	0.000	-0.009	-1.01	0.314	-0.034	-3.84	0.000	0.013	1.63	0.103
Asian, FB, 1st	-0.087	-13.21	0.000	0.004	0.69	0.492	0.006	1.05	0.292	0.048	8.79	0.000
Other & Mixed	-0.003	-0.39	0.696	0.002	0.32	0.750	-0.002	-0.35	0.726	-0.002	-0.38	0.704
**Age**	-0.002	-20.99	0.000	-0.001	-7.00	0.000	-0.001	-11.62	0.000	0.000	-3.38	0.001
**Education**												
Reference (Less than HS)												
High school or equivalent				-0.016	-4.88	0.000				-0.046	-12.82	0.000
Some college, no degree				-0.091	-24.43	0.000				-0.087	-23.18	0.000
Occupation/Vocational training (AA)				-0.037	-6.97	0.000				-0.068	-13.51	0.000
Academic associate degree				-0.115	-22.75	0.000				-0.118	-25.74	0.000
Bachelor's degree or more				-0.263	-75.56	0.000				-0.199	-55.64	0.000
**Certificate/Industry License**												
Reference (No)												
Yes				0.005	2.33	0.020				0.010	4.87	0.000
**Region**												
Reference (Northeast)												
Midwest				0.007	2.35	0.019				0.007	2.51	0.012
South				0.002	0.59	0.554				-0.001	-0.43	0.664
West				-0.008	-2.76	0.006				-0.005	-1.72	0.086
**Health Status**												
Reference (Excellent/Very Good/Good)												
Fair/Poor Health				0.017	4.49	0.000				0.009	2.67	0.008
**Marital Status**												
Reference (All Married)												
Widowed/Divorced/Seperated				0.022	7.01	0.000				0.013	5.41	0.000
Never Married				0.018	7.62	0.000				0.019	8.29	0.000
**Constant**	0.677	130.84	0.000	0.716	113.68	0.000	0.522	115.51	0.000	0.593	98.39	0.000
Number of Cases	46,295						43,360					

Online Resource Table S5 Regression Results with Vibration Index as Dependent Variable

Vibration Index	Male Unadjusted			Male Adjusted			Female Unadjusted			Female Adjusted		
	**Coefficient**	**t**	**P>t**	**Coefficient**	**t**	**P>t**	**Coefficient**	**t**	**P>t**	**Coefficient**	**t**	**P>t**
**Race, Ethnicity & Immigration Gen**												
Reference (Latino, USB, 3rd+)												
Latino, USB, 2nd	0.009	1.70	0.089	0.011	2.17	0.030	0.000	0.13	0.896	0.000	-0.01	0.992
Latino, FB, 1st	0.081	18.47	0.000	0.055	13.15	0.000	0.025	14.21	0.000	0.019	10.64	0.000
NH White	-0.017	-4.55	0.000	0.003	0.84	0.402	0.000	-0.04	0.969	0.003	2.37	0.018
NH Black	-0.008	-1.73	0.084	-0.003	-0.74	0.458	0.006	3.68	0.000	0.007	4.36	0.000
Asian, USB, 3rd +	-0.046	-4.14	0.000	-0.005	-0.46	0.644	-0.006	-1.53	0.127	-0.002	-0.50	0.615
Asian, USB, 2nd	-0.070	-8.91	0.000	-0.020	-2.76	0.006	-0.009	-2.85	0.004	-0.004	-1.22	0.221
Asian, FB, 1st	-0.072	-13.65	0.000	-0.024	-4.80	0.000	-0.002	-0.92	0.359	0.002	0.96	0.335
Other & Mixed	0.005	0.82	0.412	0.008	1.52	0.130	0.004	1.87	0.061	0.004	1.84	0.066
**Age**	0.000	3.24	0.001	0.000	3.29	0.001	0.000	1.18	0.238	0.000	3.28	0.001
**Education**												
Reference (Less than HS)												
High school or equivalent				-0.008	-2.69	0.007				-0.011	-8.44	0.000
Some college, no degree				-0.061	-19.24	0.000				-0.018	-13.34	0.000
Occupation/Vocational training (AA)				-0.029	-6.50	0.000				-0.022	-11.75	0.000
Academic associate degree				-0.082	-18.96	0.000				-0.024	-14.39	0.000
Bachelor's degree or more				-0.152	-51.10	0.000				-0.028	-21.11	0.000
**Certificate/Industry License**												
Reference (No)												
Yes				0.023	11.45	0.000				-0.005	-6.25	0.000
**Region**												
Reference (Northeast)												
Midwest				0.004	1.56	0.118				0.003	3.08	0.002
South				0.011	4.44	0.000				0.001	0.99	0.321
West				0.003	1.06	0.291				0.004	3.71	0.000
**Health Status**												
Reference (Excellent/Very Good/Good)												
Fair/Poor Health				0.003	0.99	0.323				0.000	0.11	0.912
**Marital Status**												
Reference (All Married)												
Widowed/Divorced/Seperated				0.005	1.88	0.060				0.003	3.39	0.001
Never Married				-0.021	-10.31	0.000				0.001	1.48	0.139
**Constant**	0.137	33.06	0.000	0.188	34.82	0.000	0.027	17.49	0.000	0.041	18.45	0.000
Number of Cases	46,295						43,360					

Online Resource Table S6. Regression Results with Repetition Index as Dependent Variable

**Repetition Index**												
	Male Unadjusted			Male Adjusted			Female Unadjusted			Female Adjusted		
	**Coefficient**	**t**	**P>t**	**Coefficient**	**t**	**P>t**	**Coefficient**	**t**	**P>t**	**Coefficient**	**t**	**P>t**
**Race, Ethnicity & Immigration Gen**												
Reference (Latino, USB, 3rd+)												
Latino, USB, 2nd	-0.003	-0.54	0.586	-0.003	-0.73	0.463	-0.005	-0.86	0.389	-0.006	-1.31	0.190
Latino, FB, 1st	0.048	12.42	0.000	0.028	7.52	0.000	0.084	18.29	0.000	0.059	13.64	0.000
NH White	-0.038	-11.82	0.000	-0.016	-5.29	0.000	-0.028	-7.90	0.000	-0.006	-1.69	0.092
NH Black	0.009	2.31	0.021	0.011	2.94	0.003	-0.005	-1.15	0.251	-0.003	-0.81	0.421
Asian, USB, 3rd +	-0.031	-3.11	0.002	0.006	0.60	0.550	-0.044	-4.07	0.000	-0.002	-0.21	0.837
Asian, USB, 2nd	-0.044	-6.40	0.000	-0.003	-0.50	0.617	-0.023	-2.86	0.004	0.020	2.65	0.008
Asian, FB, 1st	-0.030	-6.51	0.000	0.019	4.40	0.000	-0.008	-1.55	0.121	0.029	5.87	0.000
Other & Mixed	0.001	0.16	0.869	0.004	0.73	0.463	-0.003	-0.54	0.590	-0.002	-0.45	0.652
**Age**	-0.001	-27.66	0.000	-0.001	-10.88	0.000	0.000	-6.21	0.000	0.000	1.10	0.270
**Education**												
Reference (Less than HS)												
High school or equivalent				-0.016	-6.18	0.000				-0.020	-6.06	0.000
Some college, no degree				-0.052	-18.94	0.000				-0.045	-13.22	0.000
Occupation/Vocational training (AA)				-0.039	-10.03	0.000				-0.036	-7.78	0.000
Academic associate degree				-0.067	-17.77	0.000				-0.074	-17.70	0.000
Bachelor's degree or more				-0.143	-55.09	0.000				-0.152	-46.83	0.000
**Certificate/Industry License**												
Reference (No)												
Yes				-0.005	-3.09	0.002				-0.026	-14.05	0.000
**Region**												
Reference (Northeast)												
Midwest				0.000	0.01	0.988				0.001	0.25	0.803
South				-0.005	-2.37	0.018				-0.002	-0.73	0.464
West				-0.009	-4.09	0.000				-0.003	-1.28	0.202
**Health Status**												
Reference (Excellent/Very Good/Good)												
Fair/Poor Health				0.009	3.28	0.001				0.006	2.10	0.036
**Marital Status**												
Reference (All Married)												
Widowed/Divorced/Seperated				0.017	7.33	0.000				0.010	4.32	0.000
Never Married				0.026	14.20	0.000				0.013	6.20	0.000
**Constant**	0.578	158.17	0.000	0.592	125.60	0.000	0.518	126.2	0.000	0.568	103.74	0.000
Number of Cases	46,295						43,360					
