# Supplementary material for: Racial, Ethnic, and Immigrant Generational Disparities in Physically Strenuous and Hazardous Work Conditions
Source: J Immigr Minor Health. Author manuscript; Available in PMC 2024 Apr 1. (PMC10937783; doi:10.1007/s10903-023-01552-8)
Supplement: Supp 2 [file NIHMS1944658-supplement-Supp_2.docx]

**Online Resource 1**

**Paper Title:** “Racial, Ethnic, and Immigrant Generational Disparities in Physically Strenuous and Hazardous Work Conditions”

**Journal:** *Journal of Immigrant and Minority Health*

**Caption**: This file contains the wording of the O*NET questions and answers for each of the items included in the indices of physical activity used in the paper.

**Questions on Each Type of Physical Activity from the O*NET Work Context and Work Activities Questionnaires**

**Source:** O*NET Resource Center, U.S. Department of Labor, Work Context and Work Activities Questionnaires. Available at: <https://www.onetcenter.org/questionnaires.html>. Accessed July 3, 2023.

**General Physical Activity**

- **General occupational physical activity – 4 items**

1. What level of PERFORMING GENERAL PHYSICAL ACTIVITIES is needed to perform *your current job*?

1 – Walk between workstations in a small office

2 –

3 –

4 – Paint the outside of a house

5 –

6 – Climb up and down poles to install electricity


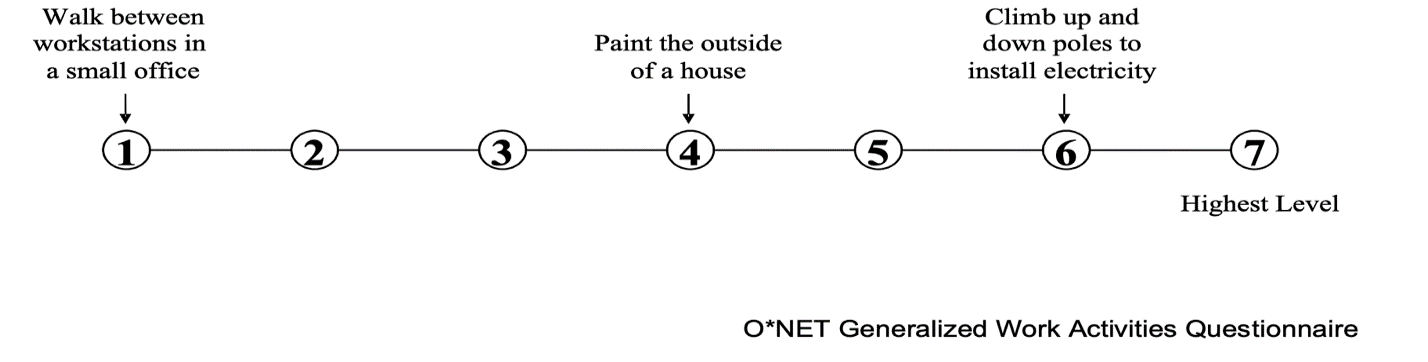
7 – Highest Level

2. How important is PERFORMING GENERAL PHYSICAL ACTIVITIES to the performance of *your current job*?

1 – Not Important

2 – Somewhat Important

3 – Important

4 – Very Important

5 – Extremely Important

3. How much time in your current job do you spend walking or running?

1 - Never

2 – Less than half the time

3 – About half the time

4 – More than half the time

5 - Continually or almost continually

4. How much time in your current job do you spend keeping or regaining your balance?

1 - Never

2 – Less than half the time

3 – About half the time

4 – More than half the time

5 - Continually or almost continually

- **Hazardous Conditions – 6 items**

1. How often does your current job require that you be exposed to high places? This can happen for workers who work on poles, scaffolding, catwalks, or ladders longer than 8 feet in length.

1- Never

2 - Once a year or more but not every month

3 - Once a month or more but not every week

4 - Once a week or more but not every day

5 – Every day

2. How often does your current job require that you be exposed to hazardous conditions? This can happen when working with high voltage electricity, flammable material, explosives, or chemicals. Do not include working with hazardous equipment.

1- Never

2 - Once a year or more but not every month

3 - Once a month or more but not every week

4 - Once a week or more but not every day

5 – Every day

3. How often does your current job require that you be exposed to hazardous equipment? This includes working with saws, close to machinery with exposed moving parts, or working near vehicular traffic (but not including driving a vehicle).

1- Never

2 - Once a year or more but not every month

3 - Once a month or more but not every week

4 - Once a week or more but not every day

5 – Every day

4. In your current job, how often are you exposed to contaminants (such as pollutants, gases, dust, or odors)?

1- Never

2 - Once a year or more but not every month

3 - Once a month or more but not every week

4 - Once a week or more but not every day

5 – Every day

5. How often does your current job require that you be exposed to radiation?

1- Never

2 - Once a year or more but not every month

3 - Once a month or more but not every week

4 - Once a week or more but not every day

5 – Every day

6. In your current job, how often are you exposed to very hot (above 90° F) or very cold (under 32° F) temperatures?

1- Never

2 - Once a year or more but not every month

3 - Once a month or more but not every week

4 - Once a week or more but not every day

5 – Every day

**MUSCULOSKELETAL-RELATED PHYSICAL ACTIVITY**

- **Posture Index – 5 items**

1. How much time in your current job do you spend standing?

1 - Never

2 – Less than half the time

3 – About half the time

4 – More than half the time

5 - Continually or almost continually

2. How much time in your current job do you spend sitting?

1 - Never

2 – Less than half the time

3 – About half the time

4 – More than half the time

5 - Continually or almost continually

3. In your current job, how often are you exposed to cramped work space that requires getting into awkward positions?

1- Never

2 - Once a year or more but not every month

3 - Once a month or more but not every week

4 - Once a week or more but not every day

5 – Every day

4. How much time in your current job do you spend kneeling, crouching, stooping, or crawling?

1 - Never

2 – Less than half the time

3 – About half the time

4 – More than half the time

5 - Continually or almost continually

5. How much time in your current job do you spend bending or twisting your body?

1 - Never

2 – Less than half the time

3 – About half the time

4 – More than half the time

5 - Continually or almost continually

- **Force Index – 1 item**

1. How much time in your current job do you spend using your hands to handle, control, or feel objects, tools, or controls?

1 - Never

2 – Less than half the time

3 – About half the time

4 – More than half the time

5 - Continually or almost continually

- **Repetition Index – 1 item**

1. How much time in your current job do you spend making repetitive motions?

1 - Never

2 – Less than half the time

3 – About half the time

4 – More than half the time

5 - Continually or almost continually

- **Vibration Index – 1 item**

1. In your current job, how often are you exposed to whole body vibration (like operating a jackhammer or earth moving equipment)?

1- Never

2 - Once a year or more but not every month

3 - Once a month or more but not every week

4 - Once a week or more but not every day

5 – Every day
